# The Future of Regenerative Medicine: Cell Therapy Using Pluripotent Stem Cells and Acellular Therapies Based on Extracellular Vesicles

**DOI:** 10.3390/cells10020240

**Published:** 2021-01-27

**Authors:** Margot Jarrige, Elie Frank, Elise Herardot, Sabrina Martineau, Annabelle Darle, Manon Benabides, Sophie Domingues, Olivier Chose, Walter Habeler, Judith Lorant, Christine Baldeschi, Cécile Martinat, Christelle Monville, Lise Morizur, Karim Ben M’Barek

**Affiliations:** 1 INSERM U861, I-Stem, AFM, Institute for Stem Cell Therapy and Exploration of Monogenic Diseases, 91100 Corbeil-Essonnes, France; mjarrige@istem.fr (M.J.); efrank@istem.fr (E.F.); eherardot@istem.fr (E.H.); smartineau@istem.fr (S.M.); adarle@istem.fr (A.D.); mbenabides@istem.fr (M.B.); sdomingues@istem.fr (S.D.); ochose@istem.fr (O.C.); whabeler@istem.fr (W.H.); jlorant@istem.fr (J.L.); cbaldeschi@istem.fr (C.B.); cmartinat@istem.fr (C.M.); cmonville@istem.fr (C.M.); 2 Université Paris-Saclay, Université d’Evry, U861, 91100 Corbeil-Essonnes, France; 3 Centre d’Etude des Cellules Souches, 91100 Corbeil-Essonnes, France

**Keywords:** pluripotent stem cells, cell therapy, extracellular vesicles, exosome, acellular therapy

## Abstract

The rapid progress in the field of stem cell research has laid strong foundations for their use in regenerative medicine applications of injured or diseased tissues. Growing evidences indicate that some observed therapeutic outcomes of stem cell-based therapy are due to paracrine effects rather than long-term engraftment and survival of transplanted cells. Given their ability to cross biological barriers and mediate intercellular information transfer of bioactive molecules, extracellular vesicles are being explored as potential cell-free therapeutic agents. In this review, we first discuss the state of the art of regenerative medicine and its current limitations and challenges, with particular attention on pluripotent stem cell-derived products to repair organs like the eye, heart, skeletal muscle and skin. We then focus on emerging beneficial roles of extracellular vesicles to alleviate these pathological conditions and address hurdles and operational issues of this acellular strategy. Finally, we discuss future directions and examine how careful integration of different approaches presented in this review could help to potentiate therapeutic results in preclinical models and their good manufacturing practice (GMP) implementation for future clinical trials.

## 1. Introduction

In the background of many diseases, the degeneration and/or dysfunction of a particular cell type affects tissues and organs, which will deteriorate over time and lose their functions. In this context, regenerative medicine offers new perspectives for pathologies that otherwise remain untreated [1,2]. Hundreds of clinical trials have been initiated in the last few years for a large panel of indications. Strategies are more and more refined to comply with large-scale productions and identify the most efficient formulation to maximize therapeutic effects [3]. Tissue engineering has recently emerged and constitutes the second generation of cell-based regenerative therapy by incorporating three-dimensional (3D) biodegradable compounds mimicking the extracellular matrix and/or multiple cell types [4,5].

Besides the therapeutic effects of cells elicited by their direct presence, some of the observed benefits are mediated by their indirect actions and, as such, may not require them [6,7]. Extracellular vesicles (EVs) have emerged as important mediators of paracrine signaling and could exert such functions [8,9,10]. Intensive research on EVs over the last half-century has led to an in-depth understanding of their origin and biological functions and has placed them at the forefront of various disease treatments.

Herein, we first review the state of the art of cell therapy and its current limitations and challenges, with a particular focus on pluripotent stem cell-derived therapeutic products. In a second part, we elaborate on functions of EVs and discuss their interest in the field of “acellular therapies” for pathological conditions that affect the eye, skin, heart and skeletal muscle. Finally, we discuss the future prospects of these therapies and their combination to potentiate therapeutic outcomes.

## 2. Regenerative Medicine: State of the Art

### 2.1. Introduction

The source material for cell and tissue-based products is a major point to consider for regenerative medicine. A prerequisite for the broad application of cell products is the production of large quantities of cells, with consistency between different batches or manufacturing processes to ensure the same quality and therapeutic efficacy to each patient.

This section will describe the numerous cell sources that may be used in cell therapy and their use in selected pathological conditions. In addition, we will discuss the transition from cell therapies formulated as a cell suspension to more complex tissue-engineered products.

### 2.2. Cell Sources

Various cell sources have been considered for cell therapy and regenerative medicine, including adult material from living donors or cadavers, fetal materials and pluripotent stem cell lines.

Cell material from adult origin can be obtained directly from patients and purified/amplified in vitro (like mesenchymal stem cells (MSCs) or epithelial cells from the skin). Such autologous approach prevents the risk of rejection. However, the manufacturing process and supply logistics of autologous cell-based therapy products are highly complicated and hinder their scale out. These limitations are particularly important when addressing widespread diseases that affect millions of patients like Age-related Macular Degeneration (AMD) or when the number of cells needed for therapy is important, as in the case of large burns. Alternatively, cells can be obtained from cadavers or, when possible, living donors. For all adult sources, donor variability—histocompatibility, age, genotype, etc.—drives cell product variability. In addition, adult cell sources have a reduced potential for expansion. This is especially true for terminally differentiated adult cell types (i.e., skeletal muscle cells) that hardly proliferate, or for organs for which there is limited access to endogenous stem cells.

Another source of materials for cell therapy is aborted fetuses. Harvested cells retain a proliferation potential that is superior to that of adult cells. Although fetus stem/progenitor cells are restricted in their potentiality, giving rise to limited cell types, some of these cells are no longer present in adult tissues and could thus be used to generate specific cell types. Nevertheless, their access is highly variable around the world, depending on the abortion law and policy in place in each country. Indeed, in some countries, it is strictly prohibited; in others, abortion is prohibited after different gestational ages, which could be restrictive. The use of human fetal tissue also raises a number of ethical considerations. Besides these restrictions, the access to fetuses at a defined gestational age is complex, as highlighted for Huntington’s disease (HD) cell therapy as an example [11]. Clinical trials have aimed to restore degenerated striatal cells in the brain of HD patients through the transplantation of fetal ganglionic eminences (GE) containing future striatal cells. In the MIG-HD clinical study, 86 fetal GE tissues were grafted into HD patients but a total of 163 surgeries were cancelled due to inadequate/insufficient donor fetal material as fetal cells should be grafted within 2 days following abortion [12].

Since their first derivation, human pluripotent stem cells (hPSCs) have been considered a promising cell source for regenerative medicine. hPSCs are self-renewable and give rise to any cell type of the human body. They can be obtained from supernumerary in vitro fertilized embryos (human embryonic stem cells or hESCs) or after the conversion of adult primary cells to pluripotency by the overexpression of a cocktail of factors (human induced pluripotent stem cells or hiPSCs) [13,14,15]. hPSCs are compatible with large-scale industrial productions in good manufacturing practices (GMP) facilities and quality controlled as any other more conventional pharmaceutical products. Whereas the majority of hPSC-based clinical trials have used hESCs up to now, the field is moving toward hiPSCs as they do not require the destruction of embryos and consequently can be used worldwide without restriction [1].

In the following section, we highlight some recent therapeutic applications of hPSC-based cell therapy products.

### 2.3. hPSC-Based Cell Therapy: Selected Examples

#### 2.3.1. Retinal Degeneration

Different causes of visual impairment, such as AMD, Retinitis Pigmentosa (RP) and diabetic retinopathy [16], are characterized by the degeneration of the light-sensitive part of the eye: the retina [17]. Depending on the nature and/or stage of the disease, different cell types are susceptible to degeneration. In particular, the retinal pigment epithelium (RPE), an essential supportive tissue of the retina affected in AMD and some forms of RP, triggers the degeneration of photoreceptors (PRs). Consequently, a specific focus has emerged to replace dead or dysfunctional RPE cells in order to prevent PR degeneration and subsequent vision loss.

In the last decade, several protocols based on spontaneous differentiation have been developed to convert hPSCs into RPE cells [7,18,19,20]. The differentiation is initiated by FGF2 withdrawal (used to maintain the pluripotency state of hPSCs). After several weeks, pigmented clusters start to appear among cultured cells. These clusters, corresponding to RPE cells, can be manually isolated and then expanded. At the end of the process, hPSC-RPE cells are banked in cryovials and can be thawed on demand. More recently, defined protocols were also described to improve the yield and size of the final cell bank through the addition of cytokines and/or small molecules [21,22,23,24]. These strategies allowed to bypass manual enrichment and critical steps of the manufacturing process could be programmed in automated cell culture systems [25,26,27].

The therapeutic potential of these hPSC-RPE cells has been evaluated after injection as a cell suspension into the subretinal space between endogenous PRs and RPE/Bruch’s membrane of a rat model of RP. All these studies demonstrated the benefit of hPSC-RPE cells with a preservation of PRs and a restoration of the visual function [18,23,28,29,30]. Recent optimizations of the final formulation with the reconstruction of an organized RPE epithelium on top of different scaffolds improved graft survival and therapeutic outcomes [18,31,32]. This formulation as an organized tissue required the development of specific devices to prepare and deliver the therapeutic product to the eye [33,34,35,36].

In the context of PR degeneration, hPSCs are able to differentiate into PRs [17] through the formation of retinal organoids [37,38,39,40]. hPSC-PRs are then enriched following dissociation and cell sorting [17,41]. First transplantations of PR cell suspensions in rodent models of retinal degeneration suggested a therapeutic effect mediated by cell integration and maturation [42]. However, further studies contradicted these first results and revealed a phenomenon of cytoplasmic material transfer with remaining PRs [43,44,45]. Retinal organoids dissected into small sections were also transplanted into the subretinal space of rodents and primates. With this grafting approach, PRs create connections and elicit some signs of visual recovery [46,47,48]. However, these sheets tend to form rosettes and PRs are transplanted with other cell types present in organoids, which limits the number of connections with the other neurons present in the retina. An improvement of the PR sheet formulation is ongoing to produce an organized PR sheet with or without a RPE epithelium [3]. In particular, polymeric 3D micro-structured scaffolds could be used to organize and structure PRs [49,50,51,52]. To overcome limitations of PR functionality caused by maturation defects, another strategy is to genetically modify hPSC-PRs with an optogene that brings light sensitivity to immature PRs [53].

#### 2.3.2. Cutaneous Wounds

Skin wounds, principally caused by traumas or thermal burns, can be self-repaired by the body [5,54]. The different stages of self-wound healing include: (1) hemostasis to stop blood loss and provide a scaffold for cell migration, (2) inflammation to eliminate pathogens and tissue debris, (3) proliferation in particular of keratinocytes to achieve wound coverage and (4) remodeling of collagens, which are secreted by dermal fibroblasts [5,55,56]. However, the healing process can be impaired by the size of the wound, environmental factors (stress, smoking, medications or recreational drugs) or genetic disorders affecting the skin or wound healing capacity [5,57].

Occlusive dressings may be applied to the wound in order to improve healing by providing a mechanical protection and a moist and clean environment that favors re-epithelialization [5,56,58]. When this is not sufficient, skin substitutes composed of allogenic or autologous cells can be obtained from the in vitro expansion of epidermal progenitors [59]. These skin substitutes may be composed of epidermal, dermal, or dermo-epidermal (composite) layers [56,57,59]. For example, sheet of autologous keratinocytes from donors are expanded in vitro to generate large epidermal sheets. However, this approach is time consuming [5,56] and, in the case of severe burns, strategies to generate ready-to-use banks of keratinocytes will improve patient care. Such objectives are achieved by the preparation of skin substitutes derived from hPSCs, which facilitate the production of large banks suitable for large-scale use and clinically compatible industrial productions.

hPSCs have been successfully differentiated into basal keratinocytes capable to form a pluristratified epidermis in vitro and in vivo in rodents [60,61,62]. However, depending on the disease context, inflammation should be controlled and angiogenesis should be promoted in order to maintain the graft. Therefore, complex tissue engineering is needed to improve graft integration and survival. Endothelial cells, fibroblast and collagen may be part of the reconstructed skin substitute to mimic the dermal layer and favor revascularization [61,63,64]. An inherent difficulty in working with several cell types is their correct spatial organization to recreate the complex skin structure to ensure functional integration of the graft. Promising results were obtained using micro-patterned 3D vascular networks with iPSC-derived endothelial cells in skin substitutes, with a better engraftment and long-term survival as well as an improved functionality when grafted in immunodeficient mice [65]. The choice of the bioink to create the 3D vasculature has also an impact on the physical properties of the reconstructed skin. Indeed, skin-derived extracellular matrix bioinks reduce the shrinkage and contraction observed with the use of collagen [66]. In addition, it favors epithelial organization and barrier function.

Nevertheless, other characteristics of the skin are still missing (i.e., melanocytes, sebaceous glands and hairiness). Different protocols based on hPSCs have recently been developed to obtain melanocytes [67,68,69] and hair follicles [70,71] but the development of clinically compatible complex skin substitutes with all appendages require further developments.

#### 2.3.3. Heart Infarction

Despite advances in interventional and medication therapies, heart failure is the first cause of human death worldwide [72]. The human cardiac muscle, or myocardium, has almost no regenerative potential compared to that of lower vertebrates, the lost tissue being replaced by a fibrotic scar [73]. Existing treatments are not curative and do not trigger heart regeneration [74]. Cell therapy may address this bottleneck through the transplantation of cells sharing the same contractile properties as heart muscle cells [74,75].

Various protocols have been developed to produce cardiomyocytes from hPSCs (hPSC-CMs) both from 3D or 2D cultures [76,77,78,79,80], some of them with a high yield and purity using bioreactors [81]. For example, hPSCs are committed towards a cardiac mesodermal lineage through exposure to BMP-2 [82]. Cardiovascular progenitor cells are then maintained to a progenitor state by inhibiting the fibroblast growth factor receptor with SU-5402 [83]. Alternatively, a temporal regulation of WNT signaling pathway (i.e., initial activation followed by inhibition) is sufficient to trigger hPSC-CM differentiation [84]. hPSC-CMs display some main characteristics of cardiomyocytes such as contractility, ion channels, calcium handling and excitation propagation [85,86].

The preclinical evaluation of hPSC-CM functionality has been conducted in rodents [87] and larger animals (pigs or primates) [88,89,90]. Cells are delivered either as a cell suspension through an intramuscular route or after tissue engineering. Following injection of allogenic PSC-CMs or hESC-CMs as cell suspensions in myocardial infarcted primates, cardiac contractile functions improved [88,89,90]. In addition, injections of PSC-CMs were shown to be superior to skeletal myoblasts or MSCs for the restoration of cardiac functions and oxygen consumption in a porcine model of ischemic injury [91]. A recent phase I clinical trial delivered epicardially hESC-CM embedded in a fibrin patch into patients with severe left ventricular dysfunction (see Section 2.4. for a more detailed description; [86]).

A major concern with the use of hPSC-CMs is the apparition of a ventricular arrhythmia probably due to immaturity and automaticity of the graft [90,92]. Indeed, differentiation protocols also give rise to nodal subtypes with automaticity-associated genes like *HCN4* susceptible to trigger pacemaker currents [93,94]. Exclusion of these cells may reduce ventricular arrhythmia [75]. Overall, hPSC-CMs delivered as a cell suspension appear functionally coupled with the host myocardium, but this observation is still debated regarding tissue-engineered hearts [75,95]. In addition, the grafted cells have limited survival in vivo. Interestingly, hPSC-CMs co-transplanted with hPSC-derived epicardial cells or MSC-loaded patches improved both graft survival (i.e., size of the graft) and maturation in rodents [96,97]. Nevertheless, studies in larger animals should confirm these results to validate long-term integration and survival of transplanted hPSC-CMs.

#### 2.3.4. Skeletal Muscle Regeneration

Muscle regeneration involves the activation of PAX7 positive quiescent satellite cells that respond to tissue injury by proliferation and differentiation to give rise to MyoD positive progenitors called myoblasts (MBs) [98]. MBs then differentiate and fuse with myofibers to regenerate the damaged muscle [99]. Despite an important regenerative potential, skeletal muscle atrophy is common following trauma or congenital muscle diseases, such as Duchenne Muscular Dystrophy (DMD), but remains an unmet medical need [100].

Upon transplantation, freshly isolated rodent satellite cells are able to regenerate chemically injured skeletal muscles that were depleted by irradiation of endogenous satellite cells [101]. The dystrophin-deficient mdx mouse model of DMD was also rescued through this strategy [102,103,104]. These results hint at the promising potential of cell therapy to tackle muscle atrophy. However, satellite cells amplified through cell culture loss their regenerative potential in vivo [103]. Thus, a renewable source of cells is required to treat patients.

Protocols have been described allowing the conversion of hPSCs into myoblasts through cytokines or small molecules exposition, recapitulating in vivo developmental cues [98,99]. Briefly, hPSCs are induced to presomitic mesoderm progenitors after activation of WNT and inhibition BMP signaling [99,105]. Then, myoblast progenitors are obtained after FGF, HGF and IGF stimulations [105]. Satellite-like cells (PAX7+ cells) represented 22% of the final cell population at 4 weeks [105,106]. These cells could then be subcultured without the loss of PAX7+ population [106]. PSC-derived satellite-like cells were able to repopulate endogenous satellite cell niche and regenerate skeletal muscles [105]. In addition, the presence of ERBB3 and NGFR surface markers allowed selective enrichment of a myogenic population with increased regenerative potential in vivo in mdx mice [107]. Therefore, cell sorting of ERBB3+ cells to enrich a myogenic cell population is suitable for cell therapy. Recently, a myogenic population was obtained after only 15 days of differentiation following CD10+/CD24- cell sorting [108]. These cells are suggested to be more homogenous compared to ERBB3+/NGFR+ and more myogenic in vivo in mdx mice [108]. Similar protocols were developed to produce large-scale banks of cryopreserved hPSC-derived myogenic progenitors (expanded for a maximum of 5 × 10^11^-fold) [109].

To date, clinically compatible protocols are still missing [98]. For volumetric muscle loss, new muscle fibers should be regenerated to reconstruct the tissue cytoarchitecture. This will require specific scaffolds [110]. In addition, supportive cells (i.e., muscle resident cells), such as endothelial cells, are necessary to ensure proper tissue vascularization [98,110]. Finally, for de novo reconstruction of muscle fibers, strategies to promote innervation should be developed [111].

### 2.4. hPSC-Based Clinical Trials

Approved in 2009 by the FDA, the first clinical trial led by Geron Corporation paved the way for the use of hESC-derived cells into the clinic [112]. The company had to fill an investigational new drug application of 22,000 pages to demonstrate the safety, functionality and quality of their hESC-derived oligodendrocyte progenitors for the treatment of spinal cord injuries. Unfortunately, during this first phase I clinical trial, only half of the patient cohort had been treated before it was halted prematurely for economic reasons [113]. Asterias Biotherapeutics (acquired later by Lineage Cell Therapeutics) pursued the development of this cell therapy in a new phase I/IIa dose escalation clinical trial and announced in 2019 that signs of motor improvements without safety concerns at 12-month were observed in the 25 treated patients [114].

Since then, a number of indications were evaluated, including eye diseases, diabetes and ischemic hearts. A recent manually curated database identified 54 planned or initiated clinical trials based on hPSCs worldwide [1]. Starting from 2018, a switch was observed regarding the source material with the number of hiPSC-based clinical trials beginning to be more important compared to that of hESC-based trials.

Most of these clinical trials focus on hPSC-derived RPE (24 clinical trials) to target macular degeneration, some forms of RP and Stargardt’s disease [1,3]. This could be explained by the easy access and available live imaging technologies, as well as low number of required cells and immune privilege of the eye. hESC-RPE cells initially injected as a cell suspension were demonstrated to be safe in a total of 38 patients [115]. The two major risks associated with hPSC therapy (i.e., teratoma formation due to residual hPSCs and cell dispersion to other organs) were not reported. hESC-RPE cells were also organized as an epithelial tissue before implantation on a synthetic [34,116] or biological scaffold [18,36]. Strikingly, one of two patients treated for a severe exudative AMD gained enough vision to be able to read again a year after the transplantation of a hESC-RPE sheet seeded on a polyester scaffold [34]. These results are encouraging but should be taken with caution due to the limited number of patients.

The first autologous hiPSC clinical trial was also conducted with hiPSC-RPE epithelial sheets to target the wet form of AMD [117]. The trial was halted after the first patient due to a Japanese regulation change leading to a switch to an allogeneic hiPSC-RPE graft strategy [3]. The only treated patient maintained her visual acuity for one year [117].

Ischemic heart disease is another critical indication with four clinical trials planned or already started [1]. A completed phase I clinical trial delivered epicardially hESC-derived cardiovascular progenitors embedded in a fibrin patch into six patients with severe left ventricular dysfunction [86]. The primary outcomes were safety through the evaluation of cardiac or off-target tumors, occurrence of arrhythmia and alloimmunization. Patients had a history of myocardial infarction at least 6 months before screening. hESC-derived cardiovascular progenitors were immunomagnetically sorted for the expression of SSEA-1, a marker of pluripotency loss and expressed the cardiac transcription factor ISL1. A nearly pure population of cardiovascular progenitor (median of 97.5%) was obtained. After a follow-up of 4-and-a-half years at maximum, no signs of tumors or arrhythmias were detected in patients [114]. An alloimmunization was detected through antibodies directed against donor cells in three patients without clinically relevant signs [86]. In fact, patients were under an immunosuppressive regimen only for one month as grafted cells are suggested to be short-lived and to act through a paracrine effect. More importantly, this trial was one of the first to suggest the safety of such cell therapy products. Since 2018, other clinical trials in China and Japan have been planned with autologous or allogenic hiPSC-derived cardiomyocytes [1].

To address type 1 diabetes, Viacyte launched four clinical trials based on hESC-derived beta-like cells delivered through subcutaneous implantation [1]. These cells, able to secrete insulin, are evaluated following subcutaneous injection in an immune-encapsulation device. No safety issues were reported in a phase I/II clinical trial of 19 implanted patients (June, 2018) [114]. In addition, the differentiation of implanted cells into insulin- and glucagon- producing cells has been reported. Concerns with the long-term cell engraftment led to an evolution of the implanted device. A new version currently under evaluation is composed of two membranes: the outer one is cell permeable to support neovascularization, contrary to the inner one, which prevents immune cell infiltration [118].

Up to now, all these clinical trials have demonstrated the safety of cell therapy products based on hPSC-derivatives. While the therapeutic application of these innovative cell products is still in its infancy, encouraging observations are also associated with some signs of efficacy. However, larger clinical trials are required to confirm the therapeutic efficacy of this strategy.

### 2.5. Limitations/Challenges

Preclinical and first clinical studies have shed light on different issues and challenges. First, immune reaction to donor cells is a critical drawback. Autologous transplantation does not need an immunosuppressive regimen and favors an optimal cell survival. However, the cost of autologous cell therapy is prohibitive [119]. The delay inherent to derivate hPSCs from a patient, to differentiate the cells to a particular cell type and finally quality control the cells to ensure safety is long. This strategy is not viable when the need for a treatment concerns millions of patients, as in AMD or in conditions that affect the heart. In contrast, allogenic cell banks allow an off-the-shelf product that could be distributed and used on demand. Cell banks could be designed large enough to treat an important number of patients at an industrial scale, reducing the cost of each graft unit. As the human leukocyte antigen (HLA) of donor is not matched to the patient, the risk of immune rejection is high and the so-called immune privilege of specific organs (i.e., central nervous system or eye) not always clearly demonstrated upon allogenic transplantation. To prevent graft rejection, an immunosuppressive regimen is required but associated side effects can be important and deleterious [120]. Current immunosuppressive protocols tested for hPSC-RPE transplantation, the most advanced hPSC-based therapy, ranged from local to systemic administration [2,34,116,121]. To bypass this, two strategies are proposed: matching the HLA between donor and recipient through multiple cell banks or to genetically engineer a hypoimmunogenic hPSC bank. Human PSC banks with known HLA could be facilitated by the identification of few selected homozygous HLA-typed donors that have the potential to match the HLA of a large part of the population in a particular country [122,123]. For example, 10 selected donors could cover nearly 40% of the UK population and 150 donors cover the majority (93%) of the population [124]. A clinical trial in Japan used one HLA-matched hiPSC-RPE cell line to treat five wet-AMD patients [125]. Transplanted cells survived at one year. The authors reported a mild immune rejection that resolved following local steroid administration. Interestingly, 18.8% of the 105 AMD patients followed in this hospital had a matched HLA with this hiPSC-RPE cell line.

Other approaches under investigation depend on genetic engineering to modulate immunogenicity of donor cells in order to generate a “universal” cell bank suitable to treat any patient, instead of multiple cell banks. For example, AAV (serotype 3B)-mediated knock-in of HLA-E at the beta-2-microglobulin (B2M) locus in B2M-/- PSCs leads to overexpression of HLA-E without surface expression of HLA-A, -B or -C. This prevents stimulation of allogenic T cells or natural killer (NK) cell mediated lysis [126]. Another approach is to knockout HLA-A and –B using CRISPR-Cas9 technology while retaining HLA-C to prevent NK mediated lysis. This approach improves donor compatibility and only 12 lines of HLA-C-retained hiPSC lines could cover 90% of the world population [127]. Alternatively, the knockout of HLA I and II was combined to the expression of immunomodulatory factors PD-L1, HLA-G, and CD47 inserted in AAVS1 locus to prevent immune responses [128]. Finally, the lentiviral-mediated overexpression of only CD47 combined with the CRISPR-Cas9 knockout of HLA I and II was found to be sufficient to generate hypoimmunogenic hiPSCs. Cardiomyocytes, endothelial or smooth muscle cells derived from these hiPSCs did not elicit immune rejection [129]. However, such genetic manipulations need a careful safety assessment of the cell bank to prevent unwanted genetic alterations and/or residual transducing vectors. In addition, the insertion of a suicide gene inducing the selective death of grafted cells upon drug supplementation may provide a safety switch against uncontrolled proliferation of hypoimmunogenic hPSCs [130]. Implementation of one of these strategies will greatly improve the benefice/risk ratio and may extend cell therapy to a larger number of patients while reducing the cost.

The formulation of cell therapy is the object of intense research for achieving optimal functionality and integration within host surrounding tissues. When cells are injected as a suspension, it could affect their survival, retention into target organ or their functionality [18,31,114]. In addition, depending on the target organ, donor hPSC-derived cells are not mature enough upon delivery and, therefore, need to complete maturation in vivo following transplantation. However, additional cues may be required, especially in a diseased tissue environment. For example, a supportive micro-structured scaffold could be associated with cells to provide a suitable environment for cell maturation or retention [3,114]. In some instances, multiple cell types may be combined to improve cell integration and functionality. This is the case when multiple cell types degenerate or when a microenvironment should be recreated [7,61,63,64,96,97,110]. Stimulation of the vascularization of the grafted hPSC-derived tissue (i.e., muscle, heart, skin) is also required for its long-term survival and integration [61,63,64,98,110,114]. First hPSC-derived clinical trials addressed the safety of “simple” systems. Additional studies are still needed to determine whether the use of more complex tissue reconstructions will go hand-in-hand with better efficiency.

Finally, cell survival is not always required to achieve functional recovery of a specific organ, suggesting that a temporary paracrine effect is sufficient to stimulate endogenous regeneration in organs that retained this potency. This is shown in the case of ischemic hearts or through the action of MSCs in acute myocardial infarction, burned skins, liver or traumatic brain injuries and many other diseases [114,131,132,133,134]. Such observations raise the question of whether cell-derived products may be sufficient for a therapeutic improvement in some specific disease conditions. Among materials released by cells, EVs hold characteristics that focus the attention of the scientific community [131,133,135].

## 3. Application of EVs in Regenerative Medicine

### 3.1. Extracellular Vesicles: Definition

**Figure 1:**
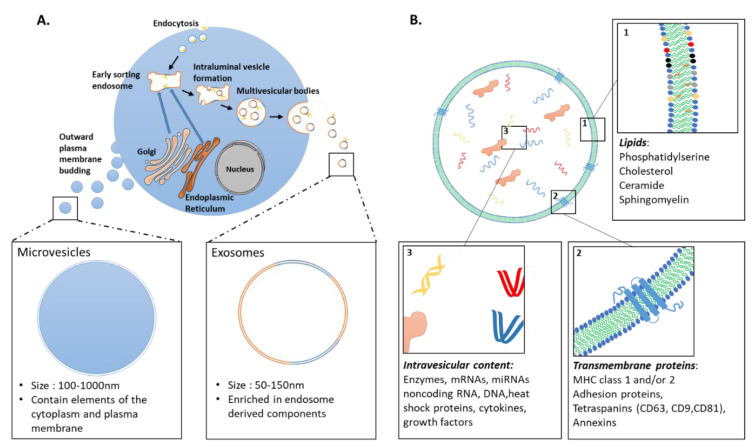
Biogenesis and general composition of EVs. (**A**) Scheme describing the biogenesis of EVs: Microvesicles are produced via the outward budding of the plasma membrane whereas exosomes arise from the fusion of multivesicular bodies with the plasma membrane. Early sorting endosomes receive materials from endoplasmic reticulum, golgi and the endocytic pathway. Multivesicular bodies are generated through the formation of intraluminal vesicles in the late sorting endosome. (**B**) Illustration of nucleic acids, proteins and lipids that can be present in EVs (this list is not exhaustive). Exact nature of EV cargo depends on the cell type, culture conditions (i.e., stress) and pathological state.

Extracellular vesicles (EVs) form a heterogeneous group of double layered lipid membrane-enclosed vesicles, with distinct biophysical properties and functions both in physiology and under pathological conditions [136]. They have emerged as important mediators of intercellular communication due to their ability to shuttle a variety of nucleic acids (including mRNAs, miRNAs), proteins, and lipids between cells (Figure 1) [137,138,139]. EVs can transmit information to target cells through different mechanisms. First, the mere interaction of EVs with surface molecules on the cell membrane can trigger intracellular signaling cascades, without delivery of their content. EVs can also fuse with acceptor cells to release their cargo, either by direct fusion with the cell membrane, phagocytosis or through a variety of endocytic pathways, including clathrin- and caveolin-mediated endocytosis, macropinocytosis, micropinocytosis, and lipid raft-mediated internalization [140,141].

EVs are categorized into three major classes based on their biogenesis: (i) Exosomes are formed by the endocytic pathway through invagination of the endosomal membrane, which ultimately forms multivesicular bodies (MVBs; Figure 1). Upon the fusion of MVBs with the plasma membrane, exosomes are released into the extracellular environment. (ii) Microvesicles are shed directly by outward vesicle budding of the plasma membrane. Exosomes are typically 50–150 nm, whereas microvesicles are 100–1000 nm in size. (iii) Apoptotic bodies (size over 500 nm) are formed by blebbing of the plasma membrane of cells undergoing apoptosis. Recently updated guidelines of the International Society for Extracellular Vesicles (ISEV) recommend use of the term extracellular vesicle as “the generic term for particles naturally released from the cell that are delimited by a lipid bilayer and cannot replicate” [142]. Unless authors are able to assign an EV to a particular biogenesis pathway, with appropriate characterization of subcellular origin, the term EV should be used exclusively. Indeed, much overlap has been present in the literature with the term “exosome” used interchangeably with “extracellular vesicle” in many studies. For the sake of clarity and coherence, we chose here to refer to them as EVs.

### 3.2. Clinical Trials Using EVs

**Figure 2:**
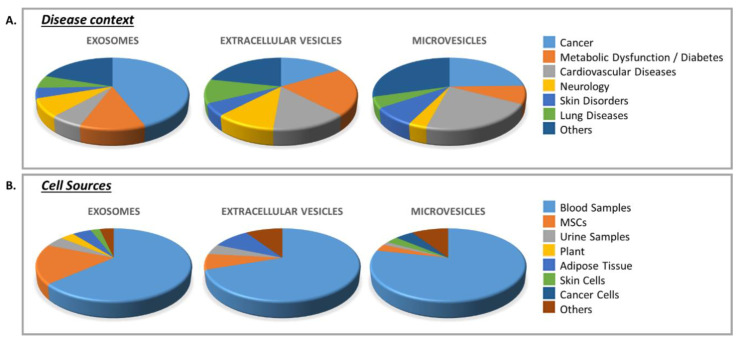
Planned or initiated clinical trials based on EVs. Diagrams representing the proportion of clinical trials targeting a group of diseases (**A**) or using a specific EV source (as biomarker or therapeutic); (**B**). These diagrams were obtained following a database search on the Clinicaltrial.gov website with indicated search terms (exosomes, extracellular vesicles, microvesicles). The majority of clinical trials use EVs as biomarkers from the blood or urine.

In recent years, a growing body of studies suggests that EVs might hold remarkable potential as therapeutics, either as active agents or delivery systems. EVs are primarily being developed as tools for anti-tumor/immunomodulatory therapies, drug delivery, and regenerative therapies [143]. In addition, EVs are extensively studied in the probing of pathophysiological states of the host as potential biomarkers in biological fluids for the diagnosis and monitoring of various diseases [144]. An increasing number of ongoing, planned or completed clinical trials have been undertaken in recent years. In order to evaluate the use of EVs in translational clinical trials, we searched for the keywords “exosomes”, “extracellular vesicles” or “microvesicles” on the ClinicalTrials.gov website. Results are presented for each EV subpopulation in relation with the disease context and source of EVs (Figure 2).

So far, most of the studies have investigated the use of EVs as reliable biomarkers in biological fluids, such as blood and urine, for diagnostic and prognostic. The immunomodulatory properties of EVs from dendritic and tumor cells are also being explored as cell-free anti-tumor agents. Promising results have been obtained in phase I and II clinical studies (reviewed in [145]). A phase II clinical trial (NCT01159288) evaluated the therapeutic potential of dendritic cell (DC)-derived EVs loaded with major histocompatibility complex (MHC) class I- and class II-restricted cancer antigens as maintenance immunotherapy after induction chemotherapy in patients bearing inoperable non-small cell lung cancer (NSCLC) [146]. This phase II trial showed that DC-derived EVs exerted NK cell effector functions in patients with advanced NSCLC, boosting the NK arm of antitumor immunity.

Recent attention has focused on the potential interest of EVs as therapeutic tools for acellular regenerative medicine. Thirty interventional clinical trials are based on the use of EVs for therapeutic purpose (Table S1). Only 16 of them are specifically evaluating the therapeutic efficacy and safety of stem-cell derived EVs in patients. Of note, none of these trials use EVs from either hPSCs or hPSC derivatives and the vast majority of them are based on MSCs. For example, a phase I clinical trial aims at studying the therapeutic potentials of condition medium from MSCs in wound healing on patients with chronic skin ulcer (NCT04134676). Similarly, a phase I/II trial uses MSC-derived EVs from normal donors to improve cutaneous wound healing of skin lesions in Epidermolysis Bullosa (EB) patients (NCT04173650). The safety and efficacy of MSC-derived EVs is also being investigated in a phase I clinical trial to promote functional recovery of large and refractory macular holes (NCT03437759). To date, none of these trials has published results yet.

### 3.3. EVs as Potential Therapeutic Tools: Selected Examples

**Figure 3:**
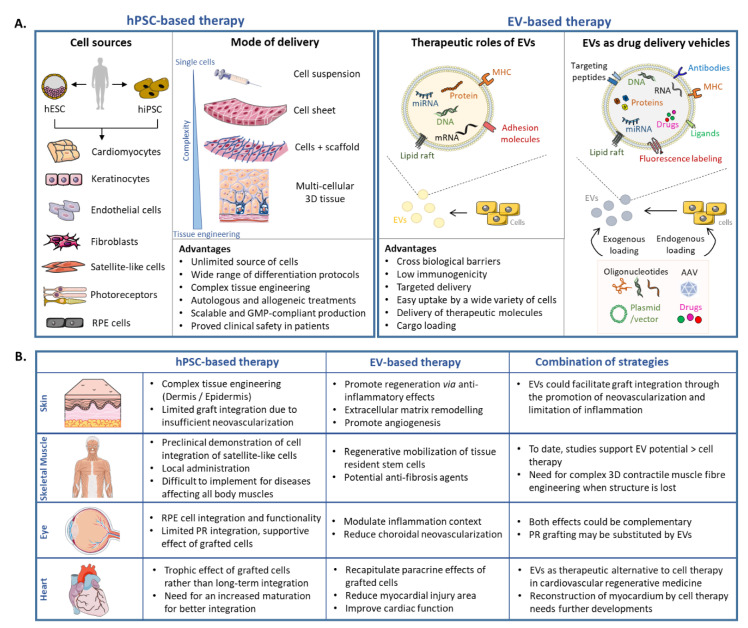
Disease-specific regenerative medicine strategy: toward tailored approaches. (**A**) Source material, mode of delivery and advantages of EV and hPSC-based therapies. (**B**) Therapeutic effects of EVs and cell therapies in preclinical animal models for selected organs (eye, skin, heart, skeletal muscle) and expected cumulative effects.

Increasing evidence suggests that EVs could recapitulate the beneficial effect of their parental cells in a number of applications. To draw a parallel with our previous section, we will focus here on the recent developments obtained with EVs as a therapeutic tool for diseases affecting the eye, skin, heart and skeletal muscle. We also explore how to integrate cell-based and acellular therapies to take advantages of these two approaches and potentiate therapeutic results (Figure 3B).

Of note, studies described herein include, but are not limited to, PSCs and derivatives. Indeed, EVs from cells with well-described paracrine actions, for example, MSCs, have been shown to exert beneficial effects in various applications. Depending on the disease and affected tissue, the therapeutic potential of EVs should therefore be examined from a variety of cell sources.

#### 3.3.1. Tissue Restoration in the Eye

Therapeutic effects of EVs have been evaluated for RP, AMD, and following injuries to the eye. Most of these studies focused on the potential of EVs to limit inflammation that would otherwise trigger the death of retinal cells [147].

As previously mentioned, different studies have started to reveal that the presence of cells might not be required to generate a beneficial effect. As an example, subretinal implantation of human neural progenitor cells preserved the vision of a model of RP through a paracrine effect [148]. The same effect is elicited by human fetal retinal progenitor cells in rats modeling RP and human patients [149,150]. As these transplantations were performed before PR degeneration, a replacement of dead cells was clearly not expected. Building on this, EVs derived from neural progenitor cells were injected subretinally in rats modeling RP before vision loss [151]. Following a single injection, the visual function and PR survival was temporarily improved (up to 28 days post-surgery). EVs were mostly internalized by Iba1+ microglial cells that had migrated from the inner retina to the subretinal space. EVs induced the downregulation of pro-inflammatory cytokines and inhibited microglia, whose suppressed activation is involved in PR survival in RP [152].

A choroidal neovascularization (CNV) is characteristic in wet AMD and can be induced in vivo with laser injuries in rodent (laser-induced CNV model). Human umbilical cord blood MSC (hUCMSC)-derived EVs injected once intravitreally were sufficient to reduce vessel leakage and the development of CNV via downregulation of *VEGF-A* [153]. Preservation of retinal functions and suppression of inflammation is equivalent when MSCs or their EVs are injected intravitreally [154]. In the same vein, retinal astroglial cell-derived EVs inhibited laser-induced CNV in mice when injected daily through the subtenon route for 7 days [155]. Interestingly, injections of EVs derived from RPE cells did not recapitulate these results [155]. Retinal neovascularization was also observed following oxygen-induced retinopathy in mice. The injection of EVs derived from hMSCs cultured under hypoxic conditions also reduced neovascularization [156].

Therefore, EVs are able to modulate retinal degeneration via the inflammatory response. This function is similar to transplanted cells, suggesting that the action of transplanted cells is likely through a paracrine effect (i.e., MSCs, retinal astroglial cells, human neural progenitors). An important point raised by these studies is that EVs or their parental cells need to be delivered at an early stage before complete degeneration. As EVs may not need complex surgeries or immunosuppression, it could be envisioned as a first line of treatment to support retinal survival and delay the requirement for a cell-based intervention. Future preclinical studies need however to determine a delivery route that allows repeated injections for long-term efficiency.

In the context of an advanced RPE cell degeneration as in late AMD, EVs may not be sufficient to recapitulate all RPE functions in order to preserve surrounding retina. Thus, endogenous RPE may be replaced through cell therapy (Figure 3A). A patch of RPE cells could be proposed as an ideal therapeutic substrate, using supporting scaffolds made of polymers or of biological composition. As EVs modulate inflammation during retinal degeneration, it should be determined whether a combined approach of RPE transplantation and EV therapy may improve visual outcomes. This is particularly true as RPE-derived EVs are not able to reduce the CNV when compared to EVs derived from paracrine-acting cells [155]. Such combined approaches may be of interest for multi-factorial diseases like AMD and may also preserve grafted cells from degeneration (Figure 3B).

Another potential EV therapy is related to PR transplantation studies for RP. Early studies showed the feasibility of transplanting post-mitotic PR precursors that achieved some degree of integration into the host mouse retina, ultimately resulting in partial visual function recovery [42,157,158,159]. The vast majority of transplanted PR precursors were later found to remain in the subretinal space where they engaged in a process of material transfer of functional proteins with host PRs [43,44,45,160,161]. The precise mechanism of this material exchange between PRs remains elusive but preclinical studies clearly demonstrated that this exchange occurs only with donor PR precursors and not with other cell types [7,160]. It could involve direct donor/host plasma membrane fusion or other methods of intercellular trafficking including EVs [162]. Therefore, it should be determined whether EVs secreted from PR precursors could recapitulate partial visual recovery obtained with PR precursors. If similar results were obtained, it would be an attractive strategy to preserve endogenous PRs without the need of cell grafting and all associated constraints (i.e., surgery, immunosuppression). When PRs have already degenerated, replacement strategies with PRs susceptible to integrate into the host retina will still be required. As discussed earlier, micro-structured scaffolds and/or addition of RPE cells could improve integration and structuration.

Taking advantage of EV ability to carry nucleic acids, they could be used as vehicles in gene therapy strategies for RP [9]. As an example, EV-associated AAV2 vector was delivered to the retina and outperformed conventional AAV2 [163]. Interestingly, these EV-AAV2 served as a robust gene delivery tool in the inner nuclear/outer plexiform and the outer nuclear layer, targeting retinal ganglion cells, bipolar cells, Müller cells as well as PRs [163]. The natural ability of EVs to deliver bioactive nucleic acids to multiple layers in the inner retina suggests that cell-free EV therapies may also benefit other traumatic or neurodegenerative ocular diseases [164].

#### 3.3.2. Cutaneous Wound Healing

Major skin injuries, resulting from extensive burns, infection or trauma, require medical interventions to heal properly [54]. Therapeutic strategies aim at facilitating the 4 phases of cutaneous wound healing–homeostasis, inflammation, proliferation, remodeling-to accelerate wound repair and regeneration [165]. Molecular and cellular events in these phases are tightly coordinated and many cell types interact with each other in a highly coordinated sequence to restore the damaged tissue [166].

EVs hold the potential to promote all phases of wound healing and facilitate skin regeneration (reviewed in [166,167]). Transition from inflammatory to proliferative phase is a key step for successful wound healing. During the early stages of inflammation, the vast majority of macrophages differentiate towards a pro-inflammatory (M1) phenotype. As the wound matures, the ratio switches to an M2 phenotype that promotes tissue remodeling and wound healing [168]. EVs obtained from lipopolysaccharide-preconditioned MSCs could convert M1 macrophage polarization to an M2 phenotype, which alleviated inflammation, and enhanced diabetic cutaneous wound healing in rats by shuttling let-7b miRNA [169]. Similarly, MSC-derived EVs promoted cutaneous wound healing in mice by regulating macrophage polarization through miR-223 [170]. In line with these results, hUCMSCs significantly decreased the number of inflammatory cells and pro-inflammatory cytokines TNF-a, IL-1, IL-6 levels while increasing the production of the anti-inflammatory cytokine IL-10 in wounds of severe burn rats [171]. The same team later found that miR-181c expression in hUCMSC-derived EVs reduced burn-induced excessive inflammation by downregulating the TLR4 signaling pathway [172].

During the proliferative phase, re-epithelization, wound contraction and angiogenesis are essential processes for the restoration of normal tissue architecture. Early recruitment of resident keratinocytes and fibroblasts is particularly important as abnormalities in the intercellular epidermal-dermal crosstalk impairs the skin repair efficiency [173]. In this context, EVs from both fetal and adult stem cell sources can improve migration and proliferation of both fibroblasts and keratinocytes [174,175,176,177,178,179,180,181,182,183]. EVs from hUCMSCs and MSCs activate signaling pathways important in wound healing, including RAC-alpha serine/threonine-protein kinase (AKT) pathway [174,177,184] and Notch signaling [182]. Increased phosphorylation of extracellular signal-regulated kinase (ERK)-1/2 [175,180,184] and inhibition of phosphatase and tensin homolog (PTEN) [184] have also been reported. Additionally, an increased extracellular matrix (ECM) deposition by fibroblasts has been observed, facilitating wound contraction. Indeed, fibroblasts increased collagen I and III production following systemic administration of MSC-derived EVs at wound sites in a mice full-thickness wound model [176]. Similar results were obtained with EVs derived from hiPSC-MSCs [185] and hUCMSCs [186]. Human adipose MSCs-derived EVs also prevented the differentiation of fibroblasts into myofibroblasts, increased the ratio of transforming growth factor-β3 (TGF-β3) to TGF-β1 and upregulated the matrix metalloproteinases-3 (*MMP3*) expression of skin dermal fibroblasts through the activation of ERK/MAPK pathway [187]. As such, EVs could be used to promote extracellular matrix remodeling and reduce scar formation.

Transplantation of cellular skin substitutes have shown considerable potential to treat both acute and chronic wounds [58]. Complex multicellular 3D models are being developed with the goal of making engineered tissues similar to their natural counterpart (Figure 3A; reviewed in [59]). However, there is still an urgent need for improving the vascularization of these substitutes to prevent necrosis and provide better long-term function and integration in clinical practice. This is doubly important as patients with chronic skin wounds usually present defects in the angiogenesis process, which consequently leads to delayed wound healing. One possibility would be the use of pre-vascularized skin substitutes that combine dermal fibroblasts, endothelial cells, and epidermal keratinocytes [63,65,66,188]. An alternative strategy would be to supplement dermo-epidermal skin grafts with EVs conveying pro-angiogenic signals to activate tissue-resident endothelial progenitor cells (Figure 3B). Indeed, exogenous EVs were shown to promote local angiogenesis in murine models of wound healing [185,189,190]. For example, EVs derived from hUCMSC, hiPSC-MSC and human urine-derived stem cells (USC) enhanced in vitro endothelial cell proliferation, migration, and tube formation [178,185,191]. HUCMSC-derived EVs promoted angiogenesis in vivo to repair deep second-degree burn injury by delivering Wnt4 and activating Wnt/B-catenin signaling in endothelial cells [191]. EVs derived from human umbilical cord blood enriched in miR-21-3p promoted the proliferation and migration of fibroblasts as well as enhanced the angiogenic activities of endothelial cells in a full-thickness skin wound mice model, thus accelerating re-epithelialization and cutaneous wound healing [184]. Similarly, EVs derived from hiPSC-MSC applied to wound sites in a full-thickness skin defect rat model promoted not only the generation of newly formed vessels, but also accelerated their maturation [185]. In another study, human USC-derived EVs markedly enhanced the generation of newly formed blood vessels in diabetic mice, in part via the transfer of pro-angiogenic protein deleted in malignant brain tumors 1 (DMBT1) [178]. Alternatively, EVs derived from hESC facilitated pressure ulcer healing by reducing endothelial senescence and promoting local angiogenesis at wound site in aged mice [190].

At present, one of the main obstacles in the treatment of skin wounds is achieving healing over time, particularly in patients with underlying skin disorders. Biomaterial-based wound dressings could be loaded with EVs to achieve sustained release to the wound sites [192,193]. For instance, Tao et al. used the polymer chitosan to prolong delivery of EVs derived from miR-126-3p-overexpressing synovium MSCs to diabetic wounds [193]. They tested this system in a diabetic rat model and found that it increased formation of granulation tissue, which provides a scaffold for the assembly of neighboring cells at wound margins, along with angiogenesis [193].

Overall, all these proofs-of-concept experiments raised considerable interest of EVs for skin repair. Of interest, their delivery to skin wounds is relatively simple due to easy access and could be sustained over time by the use of biomaterial or repeated topical applications. However, additional preclinical studies are needed to evaluate the synergic effects of combined acellular and cellular strategies.

#### 3.3.3. Heart

EVs have been investigated as promising therapeutic options for various cardiac diseases such as ischemic heart diseases and myocardial infarctions. One of the main objectives is to promote vascular repair mechanisms to reduce myocardial injury that would lead to cell death and therefore improve cardiac functions.

hESC-derived MSC conditioned medium (hESC-MSC-CoM), collected with clinically compatible processes, were shown to contain factors susceptible to modulate cardiovascular-related pathways [194]. Administration of hESC-MSC-CoM recapitulated the benefit of hESC-MSC injections in a context of post-myocardial infarction [195]. Indeed, in a porcine model of myocardial infarction, hESC-MSC-CoM intravenous treatment for 7 days enhanced capillary density, reduced the myocardial infarct size and preserved systolic and diastolic functions [195]. In addition, hESC-MSC-CoM reduced myocardial apoptosis and oxidative stress in another porcine model of ischemia and reperfusion injury [196]. This hESC-MSC-CoM contained large particles of 50–100 nm that were purified and characterized as EVs [197]. hESC-MSC-derived EVs similarly diminished the infarct size in an ex vivo mouse model of myocardial ischemia and reperfusion injury.

It was also proposed that ESC-derived EVs could stimulate endogenous myocardial regeneration [198]. Their delivery via an intramyorcardial route following mouse myocardial infarction stimulated endogenous repair (i.e., revascularization, cardiomyocyte proliferation/survival and reduced fibrosis). Interestingly, fibroblasts-derived EVs did not improve cardiac functions when compared to ESC-derived EVs in this model, highlighting differences between EV sources.

The functionality of cells from the cardiac lineage is superior to MSCs in the different heart disease models [199,200]. Therefore, EVs derived from these cells might achieve the most efficient heart recovery. Indeed, hPSC-CM-derived EVs recapitulated the therapeutic effects of their parental cells in the mouse model of chronic heart failure [201]. In this study, EVs or their parental cells were delivered once intramyocardially. Gene expression profiling identified 927 genes similarly upregulated in hearts treated with hPSC-CM-derived EVs and their parental cells as compared to control. The majority of enriched biological processes associated with these genes were predicted to improve heart regeneration and decrease fibrosis [201]. A recent study further highlighted the importance of determining which cellular source is the best candidate to produce therapeutic EVs. While both hPSC- and hPSC-CM-derived EVs protected CMs from hypoxia in vitro, only hPSC-CM-derived EVs completely improved the hypoxia-induced phenotype [202].

In order to maintain a sustained delivery, hPSC-CM-derived EVs were loaded into a collagen-based hydrogel patch [202]. Such system allows the release of EVs during 21 days in vitro. Patches loaded with EVs were implanted directly into the myocardium following an ischemic insult in a rat model of acute myocardial infarction. Rats recovered with this treatment, with improved heart contractile function and a reduction of the infarct size [202].

Overall, recent results indicate that EVs recapitulate the beneficial effects of their parental cells in the treatment of heart diseases (Figure 3B). Importantly, overall complexity associated with cell manufacturing, graft survival and patient immunosuppression are bypassed by this strategy. Future studies are nevertheless required to validate sustained EV release in large animal models as well as reproducibility across hPSC-CM-derived EV production protocols.

#### 3.3.4. Skeletal Muscle

Severe muscle injuries and genetic defects like muscular dystrophies cause myofiber death. Spontaneous reparation to regenerate skeletal myofibers do occur but are insufficient [203]. A central goal of therapeutic approaches is to re-establish the muscle structural integrity and functionality by re-populating the satellite cell niche, promoting vascularization while inhibiting fibrosis formation, and stimulating the formation of contractile muscle fibers [100]. Several cellular candidates with myogenic or non-myogenic origins have been proposed for skeletal muscle regeneration, and their transplantation has been a widely investigated therapeutic strategy [100]. However, the massive donor cell death and cellular dispersion observed after delivery of cells via injection limit their therapeutic potential. Cell therapy products are still a long way from being able to reconstruct the muscle architecture, let alone to reconstruct it with nerve and sufficient vascularization. Overall, cell therapy could be considered for small muscles but is difficult to implement for diseases affecting all body muscles.

A mounting body of evidence suggests that EVs are actively produced by skeletal muscles cells and contribute to muscle repair and regeneration [203]. For example, EVs secreted during the differentiation of human skeletal myoblasts (HSkM) into myotubes contain specific biochemical cues that promote and regulate the myogenic differentiation of human adipose-derived stem cells (HASCs) [204]. Treatment of lacerated muscle sites with these differentiating HSkM-derived EVs led to an improved muscle regeneration with a large number of regenerative myofibers associated to minimal fibrosis compared to the control group [204]. In addition to the facilitation of myofiber repair, EVs also attenuate excessive ECM deposition for optimal muscle remodeling. In muscular dystrophies and severe muscle injuries, fibrogenic cells are overactivated and hyperproliferate, leading to the substitution of skeletal muscle with nonfunctional fibrotic tissue [205]. This excessive accumulation of extracellular matrix components not only alters muscle function but also reduces the amount of tissue available for therapy and repair. Establishing new anti-fibrotic therapeutic strategies is one of the major clinical options to improve muscle function in patients. In response to hypertrophic stimuli, satellite cells give rise to myogenic progenitor cells (MPCs) able to secrete EVs containing miR-206, which represses cell collagen expression through ribosomal binding protein 1 (Rrbp1) by neighboring fibroblasts, thus preventing excessive ECM deposition [206]. Similarly, fibroblasts derived from muscle biopsies of DMD patients secreted exosomes with increased levels of miR199a-5p, causing increased fibrosis in skeletal muscle and surrounding matrix [207]. These data indicate that EVs could be of interest as potential anti-fibrotic agents.

EVs are also evaluated as potential therapeutic agents to counteract muscle wasting and skeletal muscle dysfunction. Chronic kidney disease (CKD), which ultimately leads to end-stage renal failure, often leads to muscle wasting. Intramuscular injection of EV-encapsulated miR29, previously shown to have anti-fibrotic activity, could attenuate UUO-induced body weight loss and muscle atrophy [208]. Similar results were obtained after injection of EV-miR26a in CKD mice [209]. DMD is a heritable myodegenerative disease characterized by the absence of functional dystrophin leading to progressive muscle weakness and degeneration. Recent data suggest that a treatment with EVs from cardiosphere-derived cells (CDCs) originally targeted at DMD cardiomyopathy could potentially benefit both cardiac and skeletal muscle [210]. CDC-derived EVs injected into the soleus of mdx mouse model of DMD enhanced muscle regeneration, decreased inflammation and fibrosis, allowing complete restoration of contractile forces. More surprisingly, detectable levels of full-length dystrophin were evident in the diaphragm and soleus up to three weeks after systemic CDC-derived EV delivery [210]. Dystrophin protein and transcript were undetectable in CDC-derived EVs. Moreover, analysis of exon-intron junctions for dystrophins transcripts after CDC-derived EV treatment showed no exon skipping or alternative splicing. However, RNA-seq of CDC-EVs revealed a 144-fold increase in miR-148a. Intramyocardial injection of miR-148a restored expression of dystrophin in mdx hearts 3 weeks after administration, implicating this miRNA as a potential mediator of enhanced full-length dystrophin protein synthesis [210]. Targeting EV-derived miRNAs appears as a promising strategy to improve muscle function [211].

Finally, EVs could be used as efficient delivery tools of functional cargoes in vivo to restore expression of missing proteins in patients. For example, Gao et al. demonstrated that systemic administration of exosomes loaded with CP05-conjugated dystrophin splice increased dystrophin protein expression in dystrophin-deficient mdx mice with functional improvements [212].

It has become clear that EVs enable intercellular signaling that facilitate myofiber regeneration, limit excessive ECM deposits and improve muscle functions. More strikingly, their systemic delivery improves muscle function in diseases like DMD or CDK. Future studies are now required to characterize their biogenesis, compositions and biological activities on recipient cells in both physiological and pathophysiological conditions to determine whether they might be envisioned for therapy [213].

### 3.4. Challenges

As discussed, some of the benefits observed with cell therapy are likely due to paracrine effects that can be recapitulated by EVs derived from these cells, rather than long-term engraftment and survival of transplanted cells [214]. Overall, EVs and grafted cells can elicit different outcomes according to the target organ (Figure 3B). In some instances, therapeutic effects could be similar and therefore, EV therapy may have the highest benefice/risk ratio. In other instances, both therapies are complementary and a combination of both may achieve the best therapeutic effects.

Despite their promising roles, several challenges associated with EVs as acellular therapy products still need to be overcome.

#### 3.4.1. Manufacturing EVs: Considerations

As products of cells, the identity and functions of EVs are directly correlated to their cell source. Consequently, it is essential to identify the most appropriate cell source for EV production [9]. As illustrated in Section 3.3, studies have identified a growing list of cell sources, some being more relevant than others, that could be suitable in acellular approaches for regenerative medicine. Our capacity to decipher in depth the various mechanisms by which EVs mediate biological and regenerative functions will be determinant to identify the best candidates in the future. It is also crucial to optimize the upstream processing conditions to increase the notably low EV yields [215]. Finding the optimal conditions for EV production by a specific cell type remains a challenge. Indeed, it is always a compromise between optimal conditions for growth and phenotype and those for EV production and isolation [216]. This is particularly true for the protocols that rely on the induction of a cell stress to obtain specific EVs (for example, hypoxia [156]).

Another challenge is the development of robust procedures to isolate and purify EVs with high yield and purity while preserving their structure and activity [215]. As with GMP-compliant cell manufacturing, large quantities of EVs should be produced with defined medium conditions, devoid as much as possible of xenogeneic substances and serum-derived vesicles, which otherwise have a high risk of contaminating the isolated EV sample [9]. Of note, while EVs secreted by cells cultured in serum-free conditions did not exhibit significant biophysical or size differences compared with cells cultured with serum, the expression levels of certain vesicular proteins (e.g., small GTPases, G-protein complexes, mRNA processing proteins and splicing factors) were found differentially expressed in EVs [217]. The use of commercial exosome-depleted fetal bovine serum (FBS) is another alternative to serum-free conditions. However, they are obtained by various exosome-reduction means, with each inducing their own effect upon the serum constituents and characteristics [218]. Alternatively, cells could be cultured during the EV release period with medium that has been pre-depleted of EVs [142]. However, FBS-derived EV elimination protocols have profound impacts on the cells themselves as they were shown to decrease cell growth and survival-promoting effects of FBS [219,220].

EVs are isolated from a variety of different sources, including body fluids with highly variable compositions (e.g., plasma, milk, urine, saliva) and cell culture media [221]. Importantly, the non-EV contaminants found in EV preparations differ substantially depending on their source. Specific EV isolation procedures should, therefore, be carefully determined accordingly to the starting material [221]. So far, there is no consensus on a “gold standard” technology to isolate EVs, for either therapeutic application or basic research [222,223]. Differential centrifugation/ultracentrifugation, polymer-based precipitation and density-gradient centrifugation are the most commonly used techniques but have very limited scalability and are associated with low EV recovery, disruption of EV integrity and risk of undesirable co-isolation of contaminants [9,136,223,224,225].

To purify EVs in a GMP-grade scalable manner, studies have employed ultrafiltration or tangential flow filtration (TFF) to concentrate EVs based on their size [226,227,228,229]. For example, GMP-EV-cardiac progenitor cell manufacturing was implemented for up to 8 L of conditioned medium, allowing high final product yield (≥58%) with concomitant consistent reduction of contaminants (total protein removal 97–98%) [228]. Subsequent size exclusion chromatography (SEC) allows the separation of EVs from other media components without altering their integrity [227,229,230,231]. However, size-based isolation techniques do not purify a specific EV subpopulation but rather yield complex mixtures because of the overlap in size of EVs (described in Section 3.1; [232]). Additional purification steps may also still be necessary to remove contaminants with overlapping sizes, such as bovine serum-EVs. Recently, alternative isolation techniques based on EV-surface markers have been described and seem to be promising but still in infancy [233,234]. Overall, existing techniques to isolate EVs do not have a one-size-fits-all model. The optimal isolation method(s) and acceptable level of impurity need to be carefully considered to prevent loss of function due to damage to EV-intrinsic effectors or loss of associated factors that act with EVs to exert function [142,143,221].

As with cells and other cell-derived products, maintenance of EV biological activity during storage is critical for their therapeutic use [235]. Stability should rely on the monitoring of both changes in physicochemical parameters (size, particle concentration, and morphology) and EV bioactivity. A number of studies have evaluated the impact of different storage temperatures (4, −20, and −80 °C) and repetitive free-thaw cycles on the composition and functionality of EVs isolated from various body fluids (reviewed in [235,236]). Current evidences suggest that storage at −80 °C is best-suited. Nonetheless, our current understanding of storage-mediated effects is still limited and standard criterion of EV preservation should be established in the future.

#### 3.4.2. EV-Based Therapeutics: Regulatory Aspects

Current legislation does not provide specific regulatory guidelines for EV-based therapies. In the European Union (EU) and United States of America, they enter in the framework of the biological medicinal products regulation under the definition of “biological medicine” (i.e., a medicine that contains one or more active substances made by or derived from a biological cell) [222]. The pharmaceutical classification of any biological product is determined by its active substance(s). In that regard, EV-based therapeutics are particularly challenging as it is not clear in many cases whether the biological effect depends on the vesicle internal content (EV-associated cargo), the vesicle membranes, or a combination of both [143]. As such, they share characteristics from both cell and gene therapies, which makes them difficult to classify in an existing pharmaceutical category.

Three subcategories of EV products are possible, each with specific regulatory guidance: 1/native EVs from unmodified cells, 2/EVs from genetically manipulated cells which do not contain transgene products, and 3/EVs from genetically modified cells containing transgene products [222]. In addition to the existing guidance on the manufacture of biological medicinal products, safety and quality standards for tissue- or cell-based products (DIRECTIVE 2004/23/EC and DIRECTIVE 2006/17/EC in the EU) may serve as roadmaps for EV-based therapeutics derived from human tissues and cells [222].

Stem cell therapy and tissue engineering are intimately intertwined in the development of regenerative medicine products. In recent years, research has focused on the use of biocompatible scaffolds functionalized with EVs, with or without cells, to improve regenerative capacity of the grafted products. Regulatory strategies for advanced therapy medicinal products (ATMPs)-encompassing gene therapy, somatic cell therapy, and tissue-engineered products–could be applied to such complex EV-based products (DIRECTIVE 2001/83/EC). EVs produced by genetically modified cells with biologically active transgene products could also fall within this subcategory [143]. Of note, EVs are considered as an excipient instead of an active substance when used as drug delivery systems loaded with molecules.

## 4. Conclusions

As outlined in this review, a number of key steps to develop regenerative therapies has been achieved. Preclinical evidences for many indications were the basis for first-in-man clinical trials. Main concerns related to the use of hPSCs were ruled out in clinical trials (i.e., risks of teratoma formation and cell dispersion). So far, simplified cell therapy systems have been used with some promising therapeutic results. However, preclinical evidences highlight the need for more complex cell therapy products containing various cells and/or biomaterials, structured as the native tissue. In parallel, the roles played by EVs as intercellular effectors of paracrine signaling has led to a strong interest in their use as cell-free therapeutic products to stimulate endogenous factors that would limit cell death or improve tissue regeneration. Strikingly, as these two strategies could act at different levels depending on the pathological context, their complementarity opens new perspectives to maximize therapeutic outcomes. EVs could be substituted to cell therapy in a number of indications including heart diseases, simplifying treatment complexity. However, much preclinical work is still required to characterize optimal “acellular” and cellular therapies and their potential synergistic effects. In addition, definition of appropriate conditions of GMP production need to be elaborated and customized for each application. All these future improvements may help to achieve the ultimate goal of regenerative medicine: the replacement of a degenerated tissue.

## 5. Patents

WH, CM and KB are inventors of a patent (FR3078712) related to medical devices for the preparation of retinal tissues for regenerative medicine. LM, CM and KB are inventors of a pending patent related to the automated differentiation of hPSC into RPE cells (EP3754014). LM and CB are inventors of a pending patent related to the automated differentiation of hPSC into keratinocytes (EP20305217.0). SD and CB are inventors of two pending patents related to the differentiation of hPSCs into keratinocytes (EP20305218.0) and fibroblasts (EP 20305214.7) and to the formation of a skin substitute composed of hPSCs derived cells (EP 20305213.9). AD and CB are inventors of a pending patent related to the differentiation of hPSCs into endothelial cells (EP 20305215.4).

## Data Availability

Not applicable.

## Supplementary Materials

The following are available online at https://www.mdpi.com/2073-4409/10/2/240/s1, Table S1: List of planned or initiated clinical trials using EVs as therapeutics.
